# Surface Chemistry Interactions of Cationorm with Films by Human Meibum and Tear Film Compounds

**DOI:** 10.3390/ijms18071558

**Published:** 2017-07-18

**Authors:** Georgi As. Georgiev, Norihiko Yokoi, Yana Nencheva, Nikola Peev, Philippe Daull

**Affiliations:** 1 Faculty of Physics, University of Sofia “St. Kliment Ohridski”, 1164 Sofia, Bulgaria; yana.dim.nen@gmail.com (Y.N.); peev_nikola@abv.bg (N.P.); 2 Department of Ophthalmology, Kyoto Prefectural University of Medicine, 602-8566 Kyoto, Japan; nyokoi@koto.kpu-m.ac.jp; 3 Santen SAS, 91058 Evry, France; philippe.daull@santen.com

**Keywords:** human meibum, tear film lipid layer, Novasorb technology, Cationorm, Langmuir films, dry eye, tear film

## Abstract

Cationorm^®^ (CN) cationic nanoemulsion was demonstrated to enhance tear film (TF) stability in vivo possibly via effects on tear film lipid layer (TFLL). Therefore the interactions of CN with human meibum (MGS) and TFLL in vitro and in vivo deserve special study. MGS and CN were spread at the air/water interface of a Langmuir surface balance to ensure a range of MGS/CN oil phase ratios: 20/1, 10/1, 5/1, 3/1, 2/1 and 1/1. The films capability to reorganize during dynamic area changes was evaluated via the surface pressure-area compression isotherms and step/relaxation dilatational rheology studies. Films structure was monitored with Brewster angle microscopy. CN/TFLL interactions at the ocular surface were monitored with non-contact specular microscopy. The in vitro studies of MGS/CN layers showed that (i) CN inclusion (at fixed MGS content) increased film elasticity and thickness and that (ii) CN can compensate for moderate meibum deficiency in MGS/CN films. In vivo CN mixed with TFLL in a manner similar to CN/MGS interactions in vitro, and resulted in enhanced thickness of TFLL. In vitro and in vivo data complement each other and facilitated the study of the composition-structure-function relationship that determines the impact of cationic nanoemulsions on TF.

## 1. Introduction

Caused by everyday influences like contact lens wear and extended staring at a computer screen, dry eye syndrome (DES), characterized with tear film (TF) instability, visual disturbances and chronic eye pain is today’s major ophthalmic public health disease affecting the quality of life of 10 to 30% of the human population worldwide [1]. The direct expenditures of treating a patient ($783 per year) and the burden of work productivity loss and societal impact ($11,302 per person yearly) sum up to $55.4 billion annual costof DES for the US population [2] with the impact estimated to be similar across the globe [3].

Impairment or insufficiency of any of the layers comprising the TF (tear film lipid layer (TFLL), aqueous tear (AT) or corneal epithelium surface) will result in decreased stability of TF and shorter than normal TF breakup time (TBUT). Currently meibomian gland dysfunction (MGD) resulting in quantitative and qualitative alterations of the lipid rich meibomian secretion (MGS) is considered as the world leading cause of DES with up to 86% of all DES patients showing signs of MGD [4]. As MGS is the major (>93%) component of TFLL [5,6] there is increasing recognition of the importance of oil supplementation therapies for DES treatment. Another reason for the increased pharmaceutical interest to TFLL is the much slower turnover rate of the lipid layer as compared to the AT ((0.93 ± 0.36)%/min for TFLL vs. (10.3 ± 3.7)%/min for AT [7]) showing that the tear film lipid layer is highly resistant to loss of material (i.e., to squeeze of polar lipids, PL, in the AT) during blinking. Such properties make TFLL a perspective target for drug delivery, as it may act as a natural nanoparticle that can ensure long residence time and “controlled release” of small doses of pharmaceutical ingredients [8].

Regardless of the underlying etiology, dry eye has been shown to be associated with inflammatory changes in the entire ocular surface including the adnexa, conjunctiva and cornea [9]. Since the recognition of the role of inflammation in dry eye, a number of novel treatments have been designed to inhibit various inflammatory pathways. 

Amongst the most successful therapies is the topical treatment with cyclosporine A, which due to its high hydrophobicity has to be supplemented to the eye solubilized either in oil solutions (e.g., hospital compendial formulations) or in oil-in-water (nano)emulsions in order to ensure the patient comfort and the drug bioavailability and extended residence time at the ocular surface [10,11].

The oil-in-water formulation approach has the added benefit that it can supplement oil to the TFLL and thus soothe deficiencies of the lipid layer considered as major implication in DES. Although the direct instillation of oil emollients (or other water insoluble lipophilic molecules) into tears remains commonly used approach [12], it has limited effect on the performance of TFLL in vitro and in vivo due to the low spreading and miscibility of the exogenous oils with the TFLL. However oil-in-water nanoemulsions will supplement the ocular surface not only with oils but with polar lipid like spreading agents that will enhance the oil phase uniform distribution and miscibility with TFLL across the air/tear surface [13]. This in turn can make the nanoemulsion not simply a drug vehicle for cyclosporine or other active pharmaceutical ingredient but also in perspective therapeutic formulation for DES treatment by its own self.

The first marketed ophthalmic emulsion drug product was Restasis^®^ (Allergan), a preservative-free anionic emulsion of 0.05% cyclosporine A showed to increase tear production in patients whose AT production is suppressed due to ocular inflammation. Although approved by FDA in 2002, Restasis was never accepted by European authorities.

Another approach was taken in the development of the Novasorb technology platform which resulted in the development of cetalkonium chloride (CKC) containing oil-in-water cationic nanoemulsions (CNE) Cationorm and Ikervis [11,13,14,15,16,17].

Quaternary ammoniums, such as alkyl benzyldimethylammonium chloride compounds are cationic surfactants that had attracted a lot of attention due to their preservative action [18]. However the pharmaceutical implementation of the most popular representative of this family of molecules, benzalkonium chloride (BAK; 0.01%) is decreasing due to abundance of reports showing its disruptive action on TFLL and biomembranes and its cytotoxic action [19,20,21,22]. It turned out that its acyl chain length (>95% C12, with up to 5% C14) (i) poorly matches with the long chain lipids in meibum (generally C ≥ 18) and in cell membranes (thus resulting in poor miscibility and disruption of the lipid films) and (ii) make BAK water soluble thus enhancing its diffusion through the AT and attachment to the negatively charged corneal epithelium glycocalyx. Therefore in Novasorb technology 0.002% CKC was selected as its longer C-16 tails limit the water solubility of the molecule when applied in low concentration thus ensuring the stable adsorption of CKC to the oil/water interface of the CNE nanoparticles and rendering them positive charge. The latter results in extended colloid stability of the CNE due to the electrostatic repulsion between the cationic nanoparticles [13,16]. Furthermore the longer acyl chain of CKC should ensure its better acyl chain matching and improved miscibility with TFLL thus making it more miscible with the lipid layer.

Cationorm^®^ (CN) cationic nanoemulsion (see Section 4.1 for CN composition) was shown to enhance tear film stability at the ocular surface possibly via effect on tear film lipid layer (TFLL) [23,24]. Thus the impact of CN on human meibum and TFLL in vitro and in vivo have to be studied in detail. Therefore Langmuir surface balance was used to study the interactions of Cationormwith human meibum (MGS) films at a broad range of MGS/CNE oil phase 2D ratios: 20/1, 10/1, 5/1, 3/1, 2/1 and 1/1. The films capability to reorganize during dynamic area changes were evaluated through the surface pressure-area compression/expansion isocycles. The layers dilatational rheological properties were probed via the step/relaxation method through Fourier analysis (in the 1–10^−5^ Hz range) and by exponential decay modeling of the relaxation transients [25]. This approach allows to evaluate the capability of the surface films to store energy and to recover their structure when subjected to rapid deformation as the ones at the ocular surface. Films structure was monitored with Brewster Angle microscopy. The data were compared with specular microscopy images of CN treated TFLL at the air/tear surface in vivo.

## 2. Results

### 2.1. Surface Pressure/Area Isotherms and Macroscopic Evaluation of CN/Tear Lipid Film Structure

**Figure 1:**
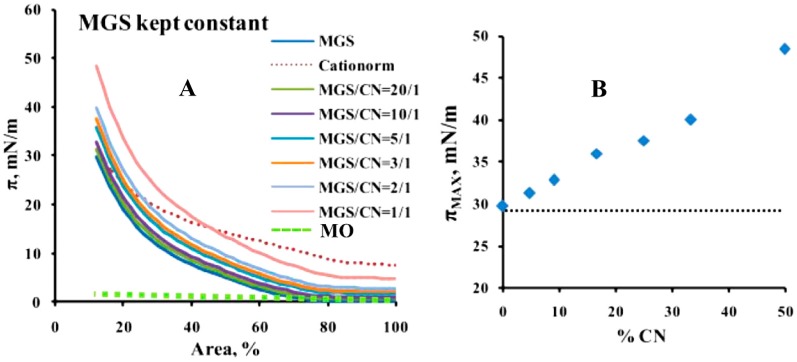
(**A**) Surface pressure area isotherms of MGS/CN pseudobinary films obtained at fixed MGS amount (i.e., the inclusion of CN increased the total lipid amount at the interface). The green dashed line shows the lack of surface activity by 30 µg of mineral oil (MO) mixture, consisting of light and heavy mineral oil in 1/1 ratio; (**B**) Dependence (blue rhombs) of maximum surface pressure, π_MAX_, on CN content at fixed MGS amount (the dashed line shows the lack of effect of MO on π_MAX_).

**Figure 2:**
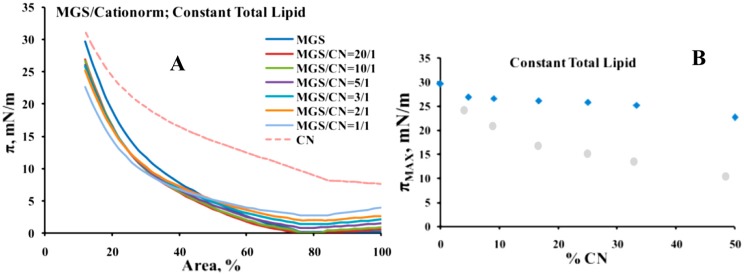
(**A**) Surface pressure area isotherms of MGS/CN pseudobinary films obtained at constant total lipid (i.e., the inclusion of CN oil was accompanied by proportional decrease of the MGS amount at the interface); (**B**) Dependence (blue rhombs) of maximum surface pressure, π_MAX_, on CN content at fixed MGS amount (the grey circles shows the inability of pure MO to assist MGS in maintenance of high value of π_MAX_).

Pseudo-binary MGS/CN films were formed at two conditions: (i) at constant amount of total lipid (i.e., CN oil phase inclusion was accompanied by proportional decrease in the human MGS quantity; Figure 1) and (ii) at constant amount of MGS (the addition of CN increased the total lipid spread at the surface; Figure 2).

When the amount of MGS was fixed, the inclusion of CN shifted the isotherms to higher surface pressures for the entire range of film areas (Figure 1A). It can be seen that the maximum surface pressure, π_MAX_, achieved at maximum degree of compression (i.e., at minimum film area) increased proportionally to the CN content, from 30 mN/m (pure MGS) to 48 mN/m (at MGS/CN = 1/1). The effect was significant (*p* < 0.01) along the entire range of compositions studied as revealed by statistical analysis (one way ANOVA with Tukey-Kramer post-hoc comparisons). In contrast control experiments with pure oil phase (dashed line in Figure 1A), showed that MO had very limited surfactant potency (reaching around 3 mN/m at compression) and showed no impact on π_MAX_. (dashed line at Figure 1B). Therefore it can be concluded that CKC and the surface active species in the aqueous phase of CN contributed to the surface activity of the cationic nanoemulsion (Figure 1B).

When the total lipid amount was kept constant (i.e., the inclusion of CN was accompanied by proportional decrease of MGS content; Figure 2), the presence of CN shifted the π/A isotherms to higher surface pressure at ≤50% film area, but at further compression the π values became lower in comparison with the pure MGS layers (Figure 2A). This is also reflected by the decrease in π_MAX_ with the increase of CN content (Figure 2B), which dropped from 30 mN/m (pure MGS) to 24 mN/m (MGS/CN = 1/1). Yet it can be seen that the drop in π_MAX_ was very moderate up to 20–30% CN content (i.e., up to 20–30% MGS depletion), with the difference between the π_MAX_ values being not statistically significant in the range of 10–25% CN (*p* > 0.5 in this composition range as revealed by one way ANOVA and Tukey-Kramer post-hoc tests). In contrast if pure MO (i.e., 1/1 *w*/*w* mixture of light and heavy mineral oil, but without CKC and rest of the surfactant ingredients of CN) was mixed with meibum then π_MAX_ dropped rapidly with the increase of MO content and the accompanying decrease in MGS part. The value of the maximum surface pressures was 30 mN/m for pure MGS and it steeply decreased with the raise of MO content dropping to 10 mN/m for the MGS/MO = 1/1 pseudobinary layers. This result is in agreement with our previous study [26], showing that squalene (an extremely hydrophobic molecule that similarly to MO displayed very low surfactant potency) contributed little to the surfactant properties of MGS layers. Thus CN was capable to compensate for partial insufficiency of human meibum at the air/water surface (in comparison to supplements consisting entirely of non-polar lipids).

**Figure 3:**
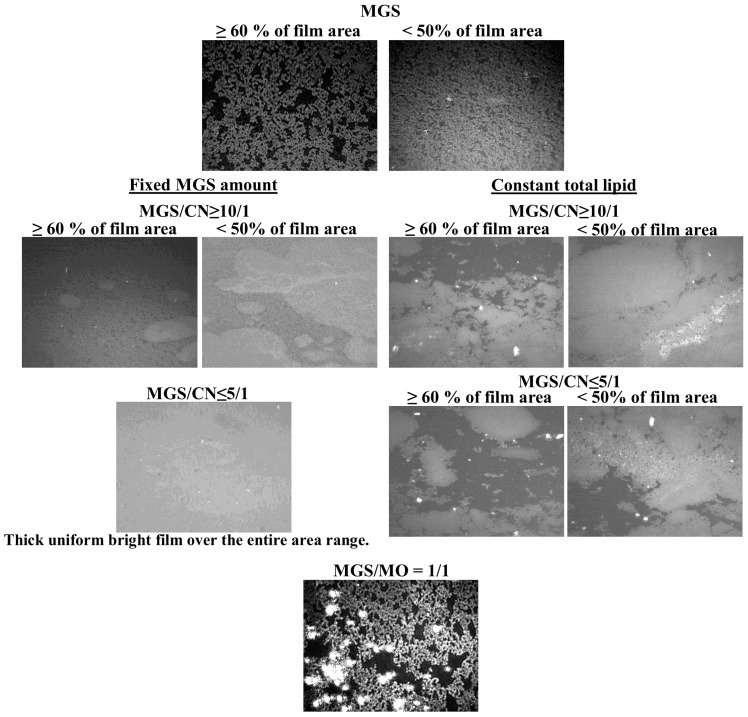
BAM micrographs of MGS and MGS/CN layers at constant amount of MGS and at fixed total lipid. BAM micrographs intensity of CN containing films was attenuated for better visual perception. (The bottom image shows the lack of spreading of MO in pseudobinary films with MGS.) (Field of view is 3000 µm × 3000 µm).

As reported in multiple studies [25,26,27,28,29] Brewster angle microscopy (Figure 3) showed that MGS alone formed heterogeneous films consisting of thin monolayer regions (dark areas) and thick aggregates of multilayer thickness (bright areas) at low (≤15 mN/m) surface pressures, and with the increase of π at further compression the thick regions enclosed to form rough and continuous multilayer while the thin monolayer regions almost disappeared. When the amount of MGS was fixed (i.e., the inclusion of CN increased the total amount of lipid at the air/saline solution surface), CN addition resulted in overall increase of the thickness (brightness) of the pseudobinary MGS/CN films and at CN/MGS ratio ≤ 5/1 relatively uniform bright layer were formed. At constant total lipid at the interface (when CN inclusion was accompanied by proportional decrease of MGS amount) CN was able to partially compensate for the MGS insufficiency at CN/MGS ratio ≥ 5/1 (as can be seen at ≤50% film area, the films were rough and continuous) but at further depletion thinner monolayer regions persisted into the pseudobinary layers even at high degree of compression.

Again the capability of CN to mix with MGS and compensate for MGS insufficiency was much better as compared to MO, where the oils just formed thick “lenses” on top of meibum (the bottom row at Figure 3) without actually affecting the amount of thin monolayer regions that were present within the film. The result is very similar to the effects of another non-surface active lipid, squalene, on MGS layer texture as shown in our previous study [26].

**Figure 4:**
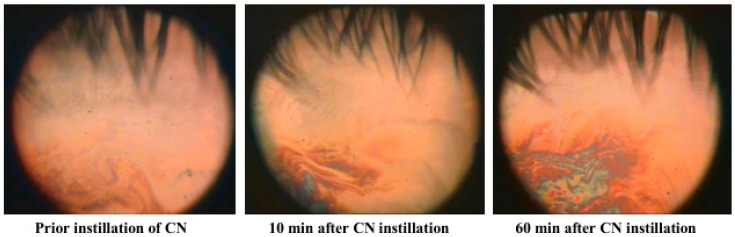
Specular microscopy images of tear film lipid layer prior and after instillation of CN at the ocular surface. (Field of view is approximately 3 cm^2^).

The in vivo specular microscopy (Figure 4) images obtained in a case study of TFLL, pure, 10 min and 60 min after CN instillation, showed that CN inclusion increased the TFLL layer thickness i.e., there was shift in the color of the TFLL image from gray-brown (≈100 nm) to a pattern of blue, green-yellow (≥180 nm). Detailed description of the interpretation of the color chart obtainable by DR-1α specular microscope can be found in Goto et al., 2003 [30]. It is remarkable that the effect was really stable in time and persisted almost unaffected for one hour after instillation. Considering the drastic difference in the turnover rate of AT (10.3± 3.7%/min) and the lipid layer (0.93 ± 0.36%/min) the durability of the CN effect indicated strong interaction of the cationic nanoemulsion with tear lipids. TFLL showed good spreading rate and interblink pattern stability prior and after treatment with Cationorm [31,32]. The movies showing TFLL behavior during blink and in open eye are provided as Supplementary Material. As CN cationic nanoemulsion is over-the-counter medical device it does not contain any active pharmaceutical ingredient. Therefore the impact of CN on TFLL is solely based on the physical interactions of the formulation with the TF. Control experiments with instillation of saline solution reveals no impact on TFLL after 10 min. The conclusions of the presented case study are supported by the results of ongoing large scale clinical study that will be reported in subsequent publication.

### 2.2. Quantitative Analysis of MGS/CN Miscibility

**Figure 5:**
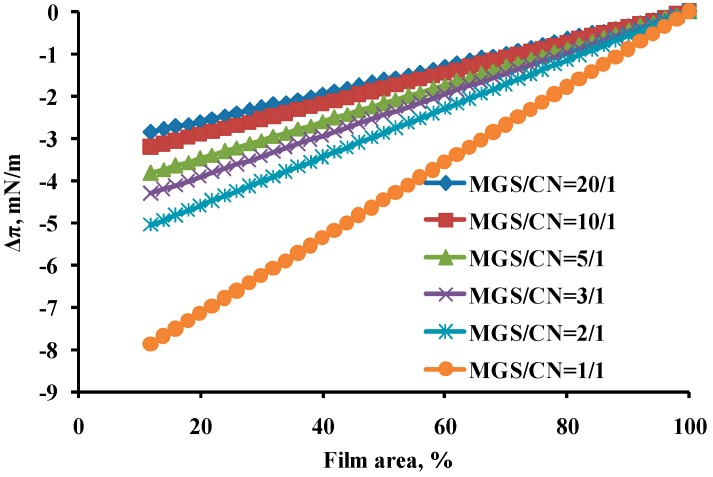
Interaction parameter Δπ calculated from the isotherms at Figure 2 using Equation (1) (see Section 4.2.1).

The π/A isotherms at Figure 2 were used to quantitatively analyze CN and MGS miscibility by Equation (1) (see Section 4.2.1) and the data are summarized in Figure 5.

It can be seen that CN and MGS displayed aggregation interaction, characterized with negative sign of theΔπ, with the term absolute value increasing (i) in the course of compression and (ii) with the CN content. For most of the tested mixtures (MGS/CN ≤ 2/1) |Δπ| ≤ 5. Only at very high CN content, MGS/CN = 1/1, Δπ reached as negative value as −8.

For pure MO it is not possible to perform such evaluation as the mineral oils mixture did not show surfactant properties, and practically had no impact on MGS surface properties. Thus it can be assumed that MO did not alter the molecular composition at the meibum/water interface and became entirely excluded in the upper non-polar lipid stratum of the MGS layer facing the air (i.e., a behavior corresponding to infinitively large negative value of Δπ).

### 2.3. Dilatational Rheology

**Figure 6:**
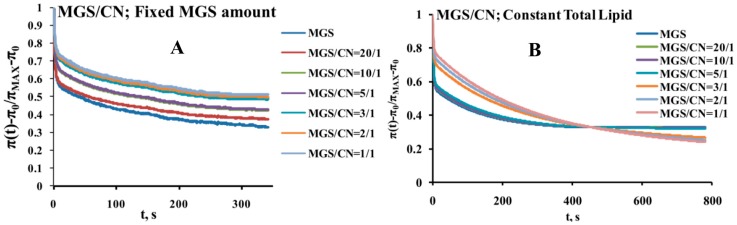
Surface pressure stress-relaxation transients of pseudo binary MGS/CN layers.

**Figure 7:**
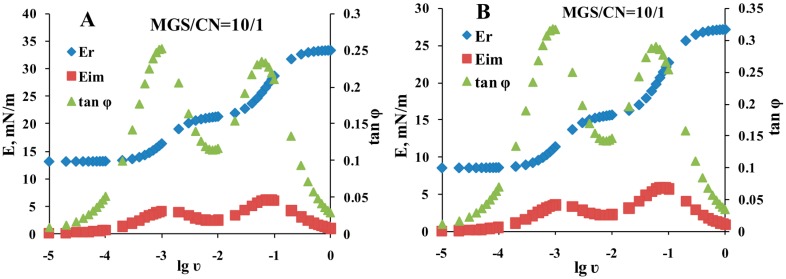
Representative Fourier transformations of typical relaxation transients. (**A**) fixed MGS amount; (**B**) constant total lipid. Data for rest of the film compositions are provided as electronic supplementary material.

When experiments were performed at fixed MGS amount, the increase in the inclusion of CN shifted the relaxations to higher surface pressures (Figure 6A). As shown by the Fourier transformation analysis this corresponds to increased contribution of the real (elastic) part of the complex modulus to the viscoelasticity of the pseudobinary films (Figure 7A and Supplementary Material).

The studies at fixed total lipid amount at the surface showed that within the studied MGS/CNE range (≥1/1) the mixed layers remained primarily elastic but at high CNE contents the relaxations shifted to slightly lower surface pressure increments (Figure 6B) and as revealed by the Fourier transformation performed by Equations (2) and (3) (Figure 7B, and supplementary information) the contribution of the viscous dissipative modulus started to increase. Still up to 20–30% CN (i.e., 20–30% MGS deficiency) the rheological properties of the pseudo-binary CN/MGS films were essentially identical to the ones of pure meibum. It can be also seen that the shape of the dependencies of the rheological parameters on frequency remained very similar thus showing the major impact of MGS for the viscoelastic behavior of the mixed layers.

**Figure 8:**
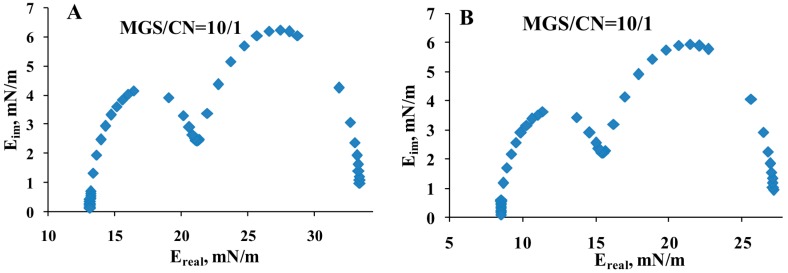
Representative Fourier transformations of typical relaxation transients ((**A**) fixed MGS amount; (**B**) constant total lipid).

The plots shown at Figure 7 were subjected to further analysis by constructing (Figure 8) their corresponding Cole–Cole plots (i.e., graph of *E*_IM_ vs. *E*_R_).

It can be clearly seen that two peaks were observed in the Cole–Cole plots, at *E*_R_ of 17 and 28 mN/m at fixed MGS amount (Figure 8A) and at *E*_R_ of 12.5 and 23 mN/m at fixed total lipid (Figure 8B).

In the context of stress relaxation the number of peaks in the Cole-Cole plot indicates the number of relaxation processes contributing to the relaxation of π [33,34,35]. It can be seen that two peaks emerged in the plots, i.e., two main processes were relevant for the relaxation kinetics. These processes can be further probed by fitting the raw relaxation transients with the corresponding number of decaying exponents and a plateau thus yielding $\Delta \pi =A_{i}\exp\sum^{\text{​}}\left(-\frac{t}{\tau_{i}}\right)+{\Delta \pi }_{\mathrm{EQ}}$, where the number of exponent terms (i) is equal to the number of peaks in the Cole–Cole plot; A-pre-exponential factor reflecting the contribution of the individual term to the relaxation; τ-characteristic relaxation time; Δπ_EQ_-plateau value.

**Figure 9:**
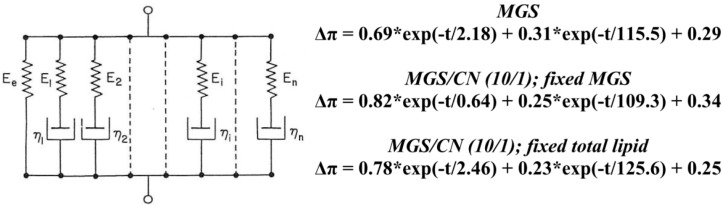
Maxwell models fit (Equation (2)) of the data presented at Figure 6.

In the framework of the generalized Maxwell model the equation represents rheological system of parallel elements in which each exponential term corresponds to a Maxwell spring-and-dashpot element and Δπ_EQ_ to a spring element denoting the equilibrium elasticity [35,36,37,38]. The relaxation time of each Maxwell element is defined as the ratio between its viscosity and elastic modulus (τ_i_ = η_i_/E_i_). The output of applying Equation (2) is summarized at Figure 9.

It can be seen that for all the films the predominant contribution (as reflected by the pre exponential term) belonged to the rapid (τ < 10 s), i.e., predominantly elastic, process.

## 3. Discussion

Human meibomian lipids consist of >90% non-polar lipids (mainly wax- and sterol esters and triacylglycerols) with limited surfactant potency and <10% polar lipids ((*O*-acyl)-omega-hydroxy fatty acids (OAHFA) and some phospholipids) of amphiphilic nature [38,39,40,41]. Due to this composition MGS forms a thick viscoelastic duplex film made of a monomolecular layer of amphiphilic polar lipids at the aqueous surface and a lipophilic suspension (comprising of lipid lamellar-crystallite aggregates immersed in a continuous disordered liquid phase) stratified on top and facing the air [42]. As this structure is very stable at compression/expansion area changes, and was found to have very long turnover rate at the ocular surface its interaction with pharmaceuticals may have long term impact on the tear film and should be carefully analyzed.

### 3.1. Interaction of CN with MGS Layers at Film Compression. Correlation with CN/TFLL Interactions In Vivo

It can be seen that when CN was added to MGS kept at constant amount (i.e., the CN inclusion elevates the total amount of lipid at the surface), it resulted in increase of the overall film thickness across the entire layer surface and enhanced surfactant properties (in terms of higher maximum surface pressure values) of the layers (Figure 1). This interaction is very different from the effect of C12 (C14)-BAK and pure oils on MGS.

As it was already shown [19,20,21,22] the penetration by С12–14 ВАK disrupted the MGS multilayers and resulted in phase separated heterogeneous layers, containing very thin monolayer patches (seen as very dark regions with BAM) and thick condensed aggregates (bright islands in BAM), with impaired mechanical properties (large π/A hysteresis, collapsibility etc.). Also С12–14 ВАK displayed significant cytotoxicity [18]. In contrast СКС containing CN mixed much better with the lipid layer and the entire film thickness across the MGS layer was increased without appearance of monolayer patches. This can be due to (i) the better acyl chain matching of the C16 CKC (in comparison to С12-ВАK) with the long chain meibomian lipids and (ii) the excess of mineral oil (СКС is merely 0.2% of the CN oil phase), which fills the gaps in the MGS layer and tightens the molecular packing density [14,15,16]. Another important factor might be the much lower concentration of СКС (0.002%) in CN, in comparison to the high amount of ВАK (0.01–0.03%) in many pharmaceuticals.

It should be also noted that CN effect on MGS is much better compared to the impact of the pure mineral oil mixture, which did not spread and mix well with the MGS film (Figure 1, Figure 2, Figure 3, Figure 4 and Figure 5). Instead the MO formed local thick spots, and essentially had no impact on the films surface pressure. The result is similar to our previous study on the interaction of squalene (hydrophobic non-surface active lipid similarly to MO) with MGS layers [26]. This big difference in the effect of CN as compared to MO can be attributed to the presence of the spreading (polar lipid like) effect of CKC and water soluble surfactants in Cationorm, which enhanced the MGS/CN interaction. The good miscibility of CN with MGS explains, the long residence time of Cationorm nanoparticles at the ocular surface (up to 8 h after instillation in rabbit eye). This in turn indicates that CN has much better therapeutic potential in the treatment of MGD and DES, than the application of entirely oil based emollients.

Therefore it is worth to discuss the possible impact of the eyedrop surface active components (CKC, Poloxamer 188 and Tyloxapol) on the interactions of CN with MGS films and how they facilitate the interplay of the formulation with the meibum layers as compared to pure MO. In a recent molecular dynamic simulation study [43] it was found that CKC stably incorporates in the TFLL at the aqueous interface, and can compensate for moderate deficiency of polar lipids. Thus it enhances the spreading and the distribution of the CN oil phase (and overall of the non-polar lipid stratum of the layers) in the pseudo-binary MGS/CN films. Due to its longer (C16) hydrophobic tail CKC exhibits better acyl chain matching [44] and miscibility with TFLL as compared with shorter chain (C12–14) alkyldimethylbenzylammonium chloride compounds [19,20]. Poloxamer 188 is found to penetrate in lipid films, but due to its hydrophilic polyoxyethylene moiety it has strong affinity to water and in contrast to CKC it readily gets squeezed out in the aqueous subphase at π ≤ 20 mN/m in experiments with phospholipid monolayers [45] and also in control experiments with MGS layers (data not shown). The interaction of tyloxapol with lipid films is studied in detail in the context of lung surfactant and it is found to fluidize the lipid films at intermediate (≤15 mN/m) surface pressures and to get squeezed out and dissolved in the aqueous subphase at further compression [46]. Thus it can be expected that P188 and tyloxapol can facilitate the miscibility of CN with TFLL at high surface area and relatively low surface pressure and then to get squeezed out of the film at further compression. Such assumption agrees with the synergetic effect of CKC and poloxamer in our studies of the interactions of cationic nanoemulsions with mebum films [47]. Similar mechanism can be assumed at the ocular surface as well. The water soluble amphiphilic substances (P188 and tyloxapol) may facilitate the miscibility of CN with TFLL after instillation in an open eye; such effect can be enhanced in dry eyes where the surface pressure (i.e., the molecular packing density) of the air/tear surface is lower as compared to normal eye (23 mN/m vs. 29 mN/m respectively) [48]. However in the course of blinking when the TFLL is subjected to drastic compression, P188 and tyloxapol are expected to become squeezed out of the TFLL in the AT and then washed out of the ocular surface by the AT turnover [49,50] while the long term impact of CN of TFLL is determined by the interaction of CKC and the formulation oil phase with TFLL. This interpretation aligns with animal model studies where it was found that the oil phase persists for hours after the eyedrop application, while the water soluble constituents of CN were rapidly washout of the ocular surface within 10 min after instillation.

The interaction of CN with MGS was reaffirmed by the good miscibility of CN with TFLL and the formation of thicker tear film lipid layer in vivo, an effect that showed remarkable durability in time and suggests the high potential of Cationorm and other Novasorb based cationic nanoemulsions to serve both as TFLL replenishing formulations and as drug delivery vehicle that can ensure long residence time of the active pharmaceutical ingredient [13,16].

These data combined with previous studies for the good tolerability of CN in cell cultures [16,17,18,19,51], suggest excellent biocompatibility and therapeutic potential of CN. The minimal to no cytotoxicity of CKC nanoemulsions (CN and Ikervis) is in stark contrast with the high damage to cell cultures treated with BAK formulations. This difference is probably due to both, (i) the very low water solubility of CKC preventing its desorption from the nanoemulsion particles into the aqueous phase (where it can diffuse to the cellular surface) and (ii) the much lower dose of CKC in CN (0.002% CKC vs. 0.01–0.03% BAK in many eyedrops).

The interactions of CN with MGS at fixed total lipid content (i.e., when the inclusion of CN is accompanied by proportional decrease of MGS amount; Figure 2) showed that although CN was not a replacement for MGS it can compensate moderate (up to 20%) MGS deficiency at the air/water interface.

### 3.2. Analysis of CN/MGS Films Viscoelasticity

Major advantage of the methodology implemented here is that the assumptions behind the stress relaxation technique and the subsequent analysis are very general [52,53], namely that (i) prior the compression step to be implemented the films are allowed to equilibrate at the chosen surface pressure and (ii) that the compression step is sufficiently small (generally implemented at less than 5%) so that linear relationship exist between the deformation and the equilibrium increment of the surface pressure. Then the stress relaxation analysis allows to study broad range of surface films as polymer mono- and multilayers, composite and heterogeneous lipid-protein mono- and multimolecular thick films and systems as complex as natural microlayers [34,35,36,37,52,53,54]. CN/MGS pseudobinary films were viscoelastic, with prevalence of the elastic part (i.e., with *E*_R_ > *E*_IM_) of the complex modulus for the entire frequency range of 10^−5^–1 Hz (Figure 6, Figure 7, Figure 8 and Figure 9). When MGS amount was fixed (i.e., the addition of CN increases the total amount of lipid at the interface), the contribution of elasticity (*E*_R_) increased with the inclusion of CN. When the experiments were performed at fixed total lipid content (i.e., when the inclusion of CN is accompanied by proportional decrease of MGS amount) at high CN concentration the contribution of elasticity decreased which can be expected due to the lower elasticity of CN compared to MGS. Still for all the compositions E_R_ remained prevalent over the viscous term (*E*_IM_). This is important as the viscoelasticity can be important parameter to distinguish in vitro between functional and “pathological” TFLL composition. Such conclusion aligns with the findings that the dilatational properties of surfactant layers, define the resistance of the air/water surface of wetting films (like the tear film) to extensional deformations (caused by capillary waves or hydrodynamic phenomena) and play a key role in the overall stability of the wetting film [55]. Indeed dilatational rheology studies [26,56] showed very different performance of (i) MGS by healthy individuals and MGD patients and of (ii) contact lens lipid extracts (CLLE) collected from Caucasians and from Asians (as a group showing higher risk for DES).

The elastic (real; storage) part, *E*_R_, of the complex modulus of “healthy” MGS and of Caucasian CLLE strongly prevails over the viscous (imaginary; dissipative) term, *E*_IM_, of the complex modulus E*. In contrast for “diseased” MGS (dMGS), *E*_IM_ > *E*_R_ in the frequency range of 10^−4^–10^−3^ Hz. As analysed in detail elsewhere [25], this means that “healthy” MGS is predominantly elastic at physiologically relevant time scale (*E*_R_ > *E*_IM_, i.e., resistant to permanent deformation), while dMGS is predominantly viscous (*E*_IM_ > *E*_R_, i.e., susceptible to deformation and less capable to prevent the extensional deformations of TF surface). In contrast to dMGS, both Caucasian and Asian CLLE were predominantly elastic for the entire frequency scale [56] which might be related with the higher content of PLs in CLLE compared to MGS. Still the elasticity of samples by Asian donors (a risk group for DES) was significantly lower compared to Caucasian lipids.

On a macroscopic level the elasticity of material can be evaluated by the ability of the sample to recover its shape after the deformation is removed. In case of TFLL this can be done by (i) accessing of TFLL spreading rate after eye opening at blink and (ii) the rate of degradation of TFLL morphology with blink [32,57,58].

It is expected that with other factors being equal, the more elastic the TFLL, the more rapidly it will spread at the air/AT surface after eye opening. TFLL spreading rate is very important clinically as during its upward movement TFLL drags the underlying aqueous tear upwards, and thus has crucial role for the uniform distribution of the AT across the ocular surface and for the stabilization of the TF [57,58,59], and avoid break formation. As revealed by Yokoi et al. [31] indeed the TFLL of DES patients spread more slowly as compared with normal subjects. A study targeting specifically the effects of TFLL abnormalities was conducted by Goto and Tseng [60], who found that there were multiple alterations of TFLL in MGD patients as compared to normals. Apart from longer spreading time (3.54 ± 1.86 s in MGD patients vs. 0.36 ± 0.22 s in healthy individuals) the pattern of spreading was different. While in healthy individuals the TFLL front progressed as uniform horizontal line across the ocular surface, in MGD eyes TFLL consisted of vertical stripes each with different spreading rate (i.e., some stripes moved faster than the others) resulting in irregular contour of the spreading front.

Another useful way to quantify the stability of TFLL is to measurehow fast the TFLL pattern deteriorates with consecutive blinks. It is expected that the more elastic the film the more stable will be its morphology. Such analysis for TFLL of MGD patients and of healthy individuals was performed by Georgiev et al., 2014 [25] who showed that the degradation of the pattern between consecutive stationary TFLL morphologies is much more pronounced in MGD eye with dMGS, compared with MGS from healthy eye. As discussed above, a more rapid deterioration of TFLL structure in vivo, suggest a stronger dissipative viscous nature of MGD lipid films compared to the more elastic lipid layer at the air/tear surface in the healthy eye. Similar trends were reported previously by Goto’s group [8,30,60]. In the case of our study the stability of TFLL morphological pattern, prior and after CN supplementation, was corresponding to the one characteristic for “healthy” tear film. This suggests that CN and Novasorb nanoemulsions may help restore the elasticity (increase *E*_R_) in dMGS and MGD patients, thus explaining their good performance [23,25] observed in the clinic for MGD and DES patients.

## 4. Materials and Methods

### 4.1. Materials

Human meibumwas collected with a platinum spatula from the lid margins of healthy volunteers working in the laboratory, three females (25–43 years old) and one male (33 years old). All volunteers gave writteninformed consent. The collection procedure was in accordance with the Declaration of Helsinki and approved by the Sofia UniversityEthical Committee (N12/10). The meibum samples were weighed, and dissolved in chloroform to a pooled stock solution with a concentration of 1 mg lipid/mL [19,20,21,22]. The experiments were performed with equiweight MGS, which based on our experien mce is well representative for the material properties of meibomian secretions collected from a variety of healthy volunteers. The standard deviation between the measured compression isotherms is less than 2%.

Cationorm (composition summarized in Table 1) was provided by Santen SAS, Evry, France.

CKC, Tyloxapol and Poloxamer P188 are pharmaceutical grade substances as defined by the European Pharmacopoeia. Poloxamer 188 has average *M*_w_ of 8400, 79.9–83.7% content of oxyethylene moieties and less than 1 ppm Ethylene oxide admixture. Tyloxapol molecular weight is variable and instead the physical properties of the substance are defined by the Pharmacopoeia standards (cloud point 94.3 °C, flash point 113 °C etc.). CKC is ≥97% purity and contains less than 3% C14 chain length admixtures. As of polydispersity the oil droplet size is within the range of 150 to 300 nm (with 80% of the droplets in the range of 200–230 nm) and the zeta potential is in the range +20 to +40 mV. (Data provided by the manufacturer). 

### 4.2. Langmuir Trough Studies

#### 4.2.1. Compression Isotherms

Surface pressure–area (π-A) isotherms were measured [26,40,61] using Langmuir surface balance µTrough XS, area 135 cm^2^, volume 100 mL (Kibron, Finland) by the Wilhelmy wire probe method (instrumental accuracy 0.01 mN/m). The trough subphase was physiological saline solution buffer (PBS, pH 7.4). Human MGS dissolved in chloroform, was deposited (30 µL of 1 mg/mL) over the air/saline surface with a microsyringe (Hamilton Co., Reno, NV, USA). When CN was added its weight concentration is evaluated via the µg of oil phase deposited at the surface. The trough was positioned under an acrylic coverto protect the surface from dust and to suppress the evaporation of thesaline solution subphase. After 15 min were given for chloroform evaporation, film compression was performed by two symmetrically moving barriers. Dynamic compression–expansion isocycling of the layer area was done with the maximum barrier’s rate (70 mm/min) at which there was no film leakage. Ten consecutive cycles were performed with each film studied. Normally after the third cycle, the shape of the π(A) curves remained constant andthose π(A) isotherms were presentedandanalyzed. All isotherms were repeated at least four times; the difference between the repetitions was less than 2%. The π(A) hysteresis was minimal between repeated isocycles of meibum films and that is why only compression isotherms are presented. The experiments were done at 35 °C. The films morphology was monitored by Brewster Angle Microscopy (MicroBAM, KSV-NIMA).

The interactions of the two components can be quantitatively evaluated bythe surface pressure increment [40,62], Δ*π*,computed as the difference between the ideal surface pressure,π_A,ideal_, (the one if the interaction between MGS and CN is additive) and the experimental value, π_A,exptl_:

Δπ = π_A,exptl_ − π_A,ideal_ (1)

Here Δπ = 0—additive MGS/CNE interaction; Δπ > 0—MGS and CNE repel each other; Δπ < 0—MGS and CNE aggregate together. The value of π_A,ideal_ is calculated as previously described for composite natural microlayers [63] by the additivity rule (π_A,ideal_ = π_A,1_ + π_A,2_) where *X*_1_ and *X*_2_ are the weight parts of MGS and CN oil respectively π_A,1_ and π_A,2_ are the surface pressure of MGS and CN film at given surface area. Strong aggregation means that CNE climbs in the non-polar lipid strata of the meibum layers, while moderate aggregation indicates more uniform mixing between CN and MGS.

#### 4.2.2. Stress-Relaxation Studies via the Small Deformations Method

In order to gather information about the dilatational viscoelasticity of meibum films, pure and with CN, the relaxation of the surface pressure was monitored after a small rapid compression deformation was applied to the surface film. Firstly the film was compressed to initial surface pressure, π_0_, of 18–20 mN/m. Then the lipid film was instantaneously and slightly contracted with a compression step, ∆*A*/*A*_0_ = 5 ± 1% (*A*_0_ is initial film area,and ∆*A*—area change). As discussed elsewhere [52,53,54] noassumptionsaremadeaboutthe surface film structure or the physicalnatureoftherelaxationprocesses (e.g., diffusion to/from the bulk solution, molecularrearrangements, exchange with secondary adsorption layers,etc.).The relaxation transient is presented in normalized coordinates (π_t_− π_0_)/(π_MAX_− π_0_) = *f*(*t*), where π_t_ is the momental value of the surface pressure and π_MAX_ is its maximal value at the start of the relaxation. The dependence of the real, *E*_R_, and imaginary part, *E*_IM_, of the complex dilatational elasticity modulus E*(*ν*) on frequency, *ν*, can be obtained via Fourier transformation, *F*, of the relaxation transients [25]:

(2) $$E^{*}\left(\nu \right)=E_{R}\left(\nu \right)+iE_{\mathrm{IM}}\left(\upsilon \right)=\frac{F\left\{d\Delta \pi \left(t\right)/dt\right\}}{F\left\{dlnA/dt\right\}}=\frac{i6.18\nu }{\Delta A/A_{0}}\overset{\infty }{\underset{0}{\int }}\Delta \pi \left(t\right)\exp\left(-i6.28\nu t\right)dt$$

Here *E*_R_ accounts for the elasticity of the surface film, while *E*_IM_ set by the product of *ν* η_d_ (η_d_ is the dilatational viscosity) accounts for the dissipative, viscous properties of the film. The number 6.28 is a brief denotement of the doubled Archimedes constant (2 × 3.14159…). The Fourier analysis of the relaxation transients was performed as previously described [52,53,64,65] utilizing commercial Fourier transform software provided by Kibron Inc. (Helsinki, Finland).

After the real and imaginary parts of the complex modulus were calculated the tangent of phase angle was computed:

(3) $$\tan\varphi =\frac{E_{\mathrm{IM}}}{E_{R}}$$

If *E*_R_ > *E*_IM_ then tan *φ* < 1 and the film is predominantly elastic. On the contrary if *E*_R_ < *E*_IM_ then tan *φ* > 1 and the film is predominantly viscous.

### 4.3. TFLL Observations

Tear film lipid layer was monitored [66] using DR-1α Video Interferometer (Kowa Company Ltd., Nagoya, Aichi, Japan) in the eye of 55 year old healthy male volunteer prior and after the instillation of Cationorm. The case study was in accordance withthe Declaration of Helsinki and approved by the Sofia University Ethical Committee (N12/10) and the volunteer provided written informed consent. The instilled drop volume was 25 µL. In animal model (depending on the species; mouse, rat, or rabbit, and the nature (efficacy or toxicity) of the studies), the instilled volume range from 3 to 50 µL. It was found that a hydrophobic tracer (solubilized in CN oil phase) remains longer on the ocular surface (up to 8–12 h in the rabbit eye), while the aqueous phase is eliminated within minutes following the instillation. The magnitude of the hydrophobic tracer signal was almost independent on the drop volume.

## 5. Conclusions

The in vitro studies of the pseudobinary layers of human meibum and Cationorm showed that (i) CN inclusion (at fixed MGS content) increased film elasticity and thickness and that (ii) CN can compensate for moderate meibum deficiency in MGS/CN films. In vivo CN mixed with tear film lipid layer in a manner similar to CN/MGS interactions registered in Langmuir surface balance experiments, and resulted in enhanced thickness of TFLL. In vitro and in vivo data complement each other and facilitated the study of the composition-structure-function relationship that determines the impact of cationic nanoemulsions on TF.

## Figures and Tables

**Table 1:** Composition of Cationorm cationic nanoemulsion.

Composition% *w*/*w* (µg/µL)	Novasorb Cationic Emulsions(Cationorm)	Function
Excipients (oil phase)		
Mineral oil (light & heavy)	0.50 (5 µg/µL)	Oily agent
	0.50 (5 µg/µL)	
Cetalkonium chloride	0.002 (0.02 µg/µL)	Cationic surfactant
Excipients (water phase)		
Tyloxapol	0.30 (3 µg/µL)	Non-ionic surfactant
Poloxamer 188	0.10 (1 µg/µL)	Non-ionic surfactant
Glycerin	1.60	Osmotic agent
Buffer system (tris-HCl/trometamine)	(0.071/0.006)	Buffer
Water for injection	q.s.*	Diluent
TOTAL	100	

* q.s.-quantum satis (until it is enough (latin)).

## Supplementary Materials

Supplementary materials can be found at www.mdpi.com/1422-0067/18/7/1558/s1.

## Abbreviations

TF	Tear film
TFLL	Tear film lipid layer
MGS	Meibomian gland secretion (or simply meibum)
CKC	Cetalkonium chloride (C16)
BAK	Benzalkonium chloride (C12)
CN	Cationorm
CNE	Cationic nanoemulsion
DES	Dry eye syndrome
AT	Aqueous tear
PL	Polar lipid
NPL	Nonpolar lipid
MO	Mineral oil
BAM	Brewster angle microscopy
