# Supplementary material for: Surface Chemistry Interactions of Cationorm with Films by Human Meibum and Tear Film Compounds
Source: Int J Mol Sci. 2017 Jul 18;18(7):1558. doi: 10.3390/ijms18071558 (PMC5536046; doi:10.3390/ijms18071558)
Supplement: Supplementary file 1 [file ijms-18-01558-s001.zip › Supplementary Files/Supplement_1_FT_relaxations_constant MGS.docx]

**Fourier analysis of the relaxation transients of MGC/CN films; MGS kept constant**

**I. Fourier analysis of the relaxation transients from MGS/CN films**
